# Rare Cutaneous Metastasis of Low-Grade Retroperitoneal Leiomyosarcoma

**DOI:** 10.7759/cureus.92362

**Published:** 2025-09-15

**Authors:** Kostandin Valle, Divya Pothuri, Sean Pirrone, Jordan Parker

**Affiliations:** 1 Dermatology, University of Missouri School of Medicine, Columbia, USA

**Keywords:** cutaneous metastasis, immunohistochemistry, low-grade retroperitoneal leiomyosarcoma, oncodermatology, rare case report, retroperitoneal tumor, skin metastasis, spindle cell neoplasm

## Abstract

Retroperitoneal leiomyosarcoma (RPL) is a rare smooth muscle malignancy that often presents silently and is typically diagnosed at an advanced stage. Cutaneous metastases from RPL are uncommon and usually indicate high-grade disease with a poor prognosis. To date, there are no published reports of low-grade RPL metastasizing to the skin. We report the case of a 55-year-old female who initially presented with intermittent abdominal pain and was diagnosed with a 5.1 x 3.1 cm retroperitoneal mass. Following surgical resection, histopathologic examination confirmed low-grade leiomyosarcoma based on spindle cell morphology and low mitotic activity. The patient recovered well postoperatively, but 18 months later, she presented to dermatology with a firm, dusky, subcutaneous nodule on her right temporal hairline. A punch biopsy confirmed cutaneous metastasis of leiomyosarcoma. A subsequent lesion in the right antecubital fossa was also biopsied, showing identical histological features. Both lesions were excised. A follow-up PET scan showed no evidence of systemic metastasis. A third nodule later developed on the right cheek, prompting oncology follow-up due to concern for deep structural involvement. This is the first reported case of a histologically low-grade RPL metastasizing to the skin. While low-grade RPLs are considered less aggressive, this case highlights their potential for metastasis. Clinicians should maintain a high index of suspicion for cutaneous metastasis in patients with RPL who develop new skin lesions, even in cases with low histologic grade.

## Introduction

Retroperitoneal leiomyosarcoma (RPL) is a rare, malignant tumor that arises from smooth muscle cells within the retroperitoneal space [1]. Due to their location, most of these tumors can grow to significant sizes while remaining clinically silent. RPLs are frequently diagnosed as high-grade tumors with a poor prognosis [1]. Diagnostic measures for RPL include computed tomography (CT) scans or magnetic resonance imaging (MRI), but confirmation of the diagnosis requires a core needle biopsy [2]. Histopathologic examination is necessary to grade the tumor. The distinction between high-grade and low-grade tumors is determined based on the number of mitotic figures per 10 high-power fields (HPF). Greater than 10 mitotic figures per 10 HPF is deemed a high-grade tumor, and less than 10 mitotic figures per 10 HPF is considered a low-grade tumor [3]. When treatment is pursued, the gold standard remains en bloc resection of the mass, often requiring resection of surrounding tissue as well [4,5]. Various studies have additionally examined the use of radiation therapy in addition to surgical resection, either preoperative, postoperative, or intraoperative, but no clinical trials have demonstrated prospective efficacy [2].

Primary cutaneous leiomyosarcomas have been recognized in the literature, often signaling a better prognosis for patients [2,6]. These are divided into dermal or subcutaneous subtypes depending on the layer of origin. In contrast, cutaneous metastasis of RPL typically signifies a high-grade tumor and favors a worse prognosis [6]. A thorough review of the literature revealed no published case reports to date of low-grade RPL metastasizing to the skin. Histopathologic evaluation of cutaneous leiomyosarcomas is congruent with increased mitotic activity and spindle-shaped cells arranged in fascicles. Immunohistochemical staining can help further confirm the diagnosis and includes stains for desmin, vimentin, smooth muscle actin, or other muscle markers [4]. The clinical presentation of cutaneous leiomyosarcoma can be variable, but often includes a novel and evolving firm nodular lesion with pigmentary changes and tenderness to palpation [4]. Here, we present a case of this phenomenon in a 55-year-old female patient.

## Case presentation

The patient first presented to the emergency department with intermittent abdominal pain in January 2023. A CT scan of the abdomen and pelvis at that time identified a right upper quadrant mass. Malignancy was suspected, and the patient was referred to surgical oncology. Surgical oncology ordered further imaging, including a repeat CT scan and an MRI. These confirmed a 5.1×3.1 cm mass adjacent to the patient's liver, which was causing a mass effect on her inferior vena cava, right adrenal gland, and liver. An exploratory laparotomy was performed in March 2023 with excision of the retroperitoneal tumor and right adrenal gland, as well as segmental resection of the suprarenal vena cava with a Dacron interposition graft. Surgical pathology reported the presence of hyperchromatic spindle-to-oval nuclei, moderate eosinophilic to clear cytoplasm, and indistinct cell borders arranged in fascicles with scattered lymphoid aggregates. There were up to seven mitotic figures per 10 HPF, and a Ki-67 demonstrated a 7% proliferative rate, both indicating low levels of cell division. As such, the tumor cells exhibited a low degree of atypia. Immunohistochemical stain was positive for desmin, caldesmon, and smooth muscle actin. This, coupled with a low degree of atypia, led to a histological diagnosis of low-grade leiomyosarcoma. The patient recovered well from the procedure and experienced no recurrence of abdominal symptoms.

**Figure 1:**
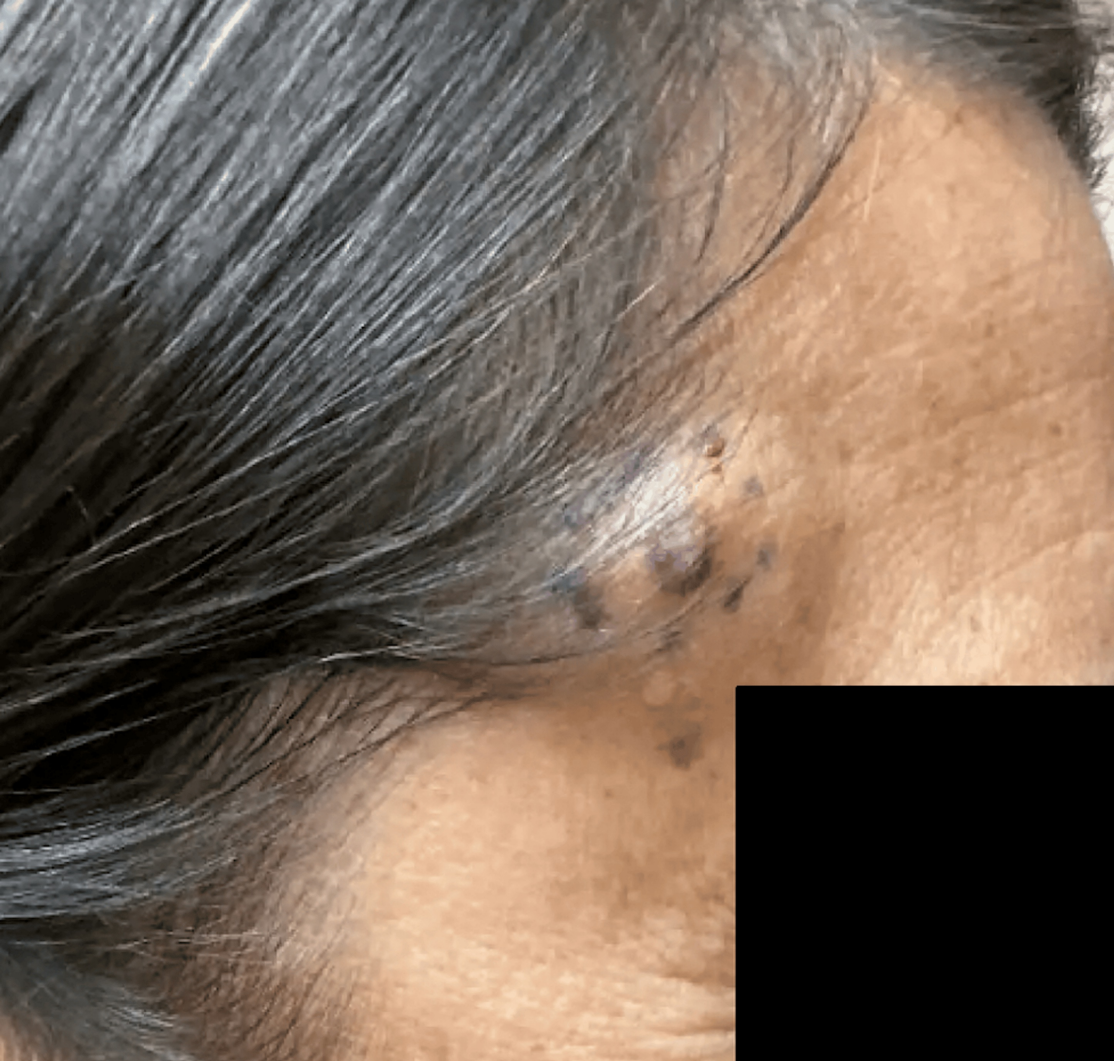
Gross clinical findings Firm, skin-colored subcutaneous nodule located on the right temple.

In November 2024, the patient presented to the dermatology clinic due to concerns of a skin lesion that had been present for an indeterminate amount of time, but was evolving. The lesion was a 1.1 cm firm, subcutaneous, slightly dusky nodule with central darkening located in the right temporal hairline, associated with pruritus and irritation (Figure 1).

**Figure 2:**
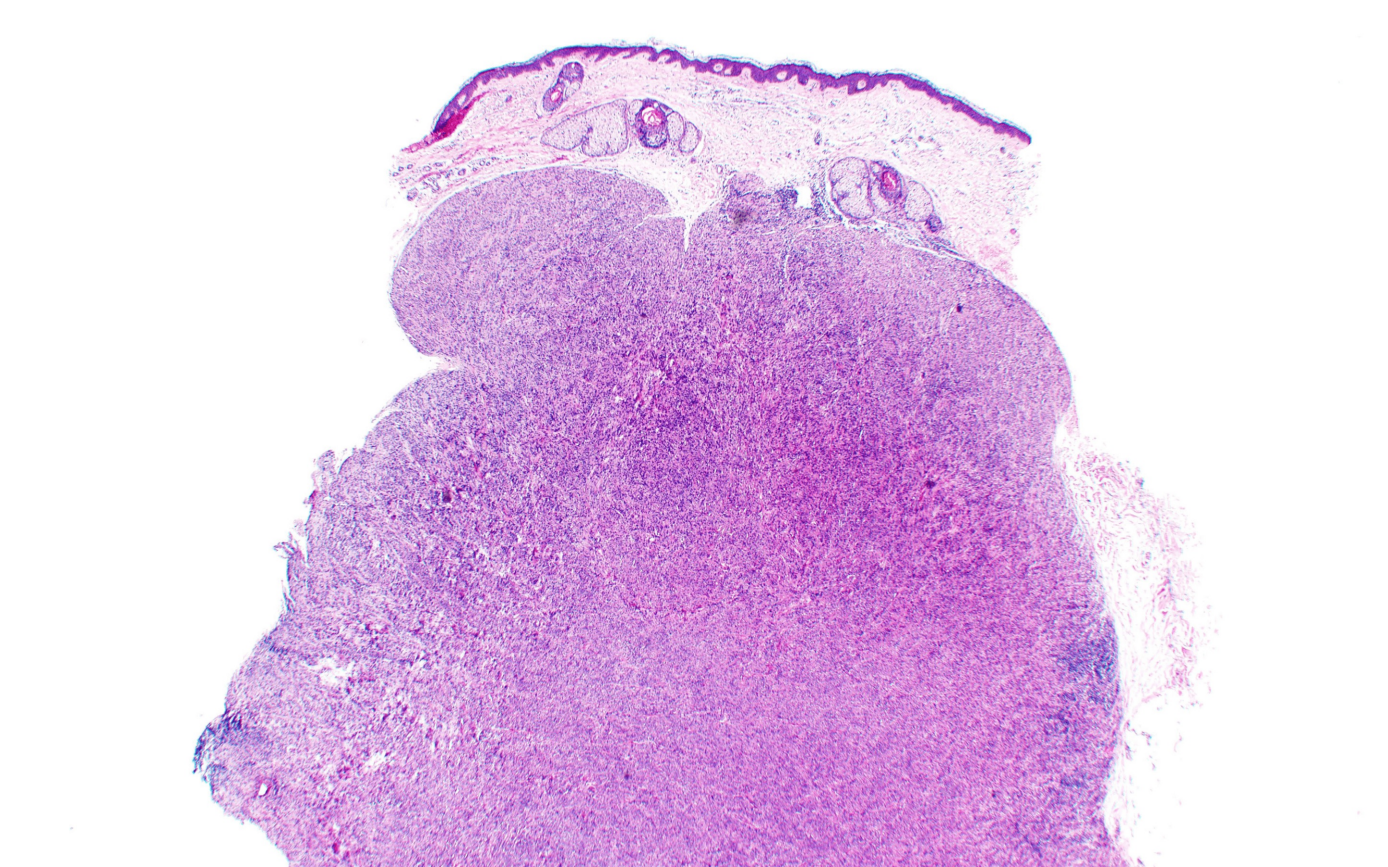
Low-power H&E stain image of the patient's metastatic lesion A punch biopsy consists of neoplastic cells with scattered lymphoid aggregates. H&E: hematoxylin and eosin

**Figure 3:**
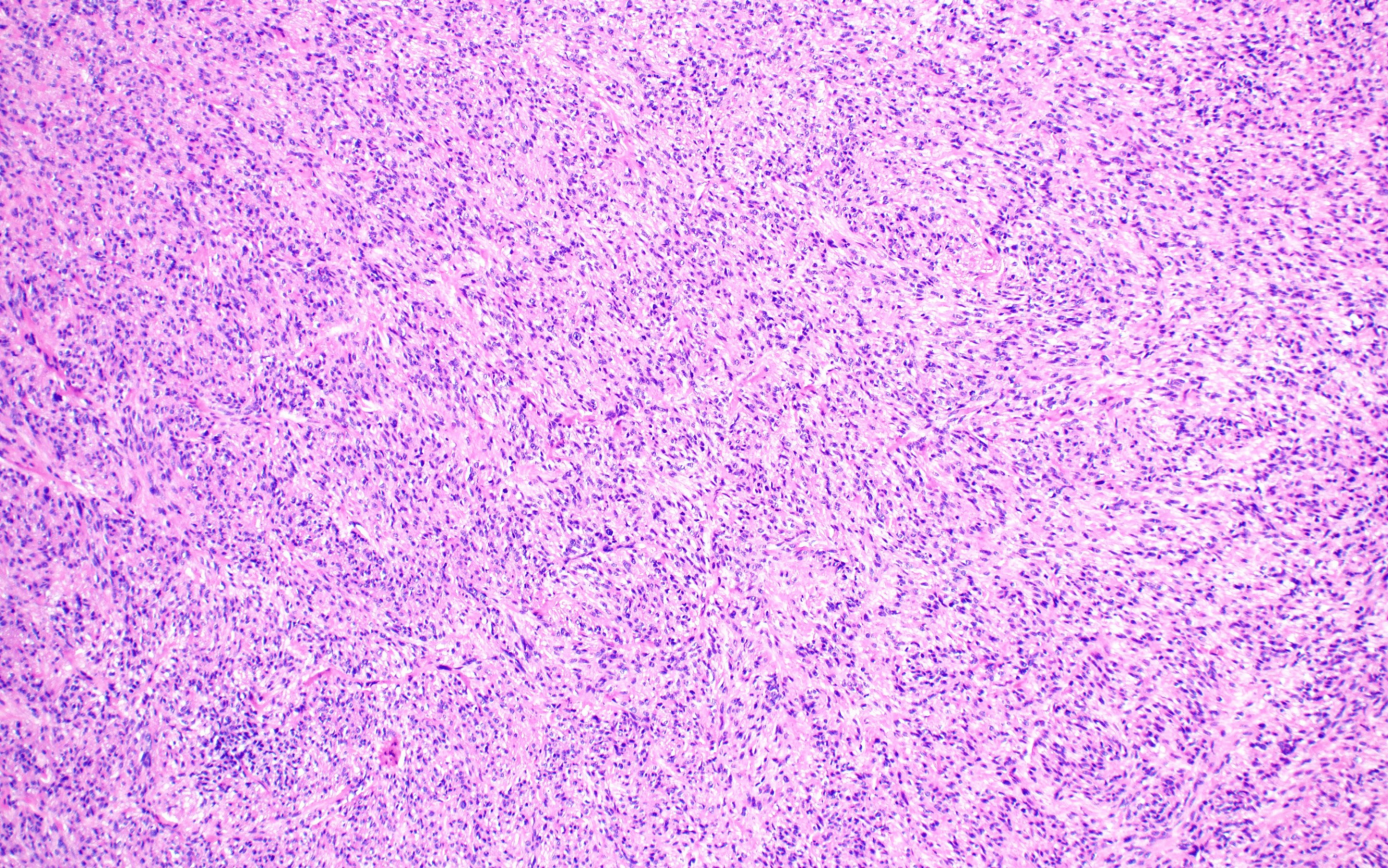
Medium power H&E stain image of the patient's metastatic lesion Neoplastic cells exhibit abundant eosinophilic activity, which helps to clear cytoplasm and lymphoid aggregates. H&E: hematoxylin and eosin

**Figure 4:**
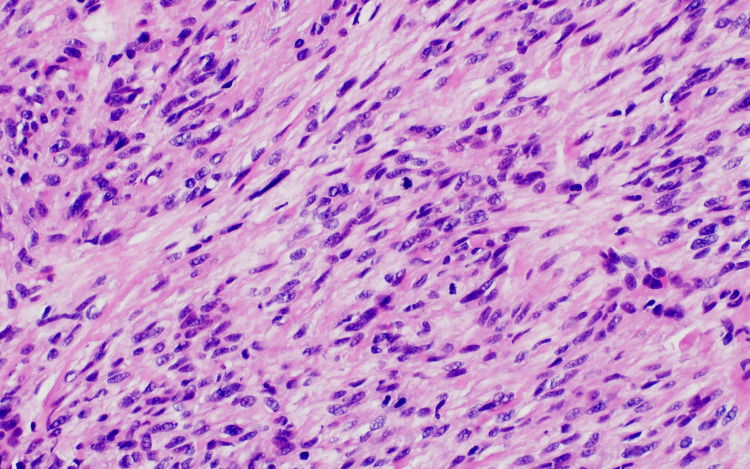
High-power H&E stain image of the patient's metastatic lesion Neoplastic proliferation composed of fascicles of spindled cells with blunt-ended, hyperchromatic nuclei and scattered mitotic figures. H&E: hematoxylin and eosin

Due to the patient's history and inconclusive presentation, a 5 mm punch biopsy was performed at this time and sent to pathology for evaluation using hematoxylin and eosin (H&E) (Figure 2). Histopathology showed proliferation composed of fascicles of spindled cells with blunt-ended nuclei, alongside neoplastic cells with hyperchromatic nuclei and scattered mitotic figures (Figures 3, 4).

The histopathological findings, confirmed by positive immunohistochemical staining for smooth muscle actin, led to a diagnosis of metastatic low-grade leiomyosarcoma. At her follow-up appointment 11 days later, the patient endorsed an additional 0.3 cm mobile subcutaneous nodular lesion in the right antecubital fossa. A 4 mm punch excision of the lesion was performed and sent to pathology, which confirmed it to be metastatic low-grade leiomyosarcoma, with identical histologic features as the first cutaneous specimen. The patient underwent excision of both cutaneous nodules, and a follow-up PET scan by oncology revealed no sign of further metastatic disease. A watchful waiting approach was recommended, as low-grade leiomyosarcoma responds poorly to systemic therapy.

At her most recent visit to the dermatology clinic in May 2025, the patient endorsed a new lesion on her right cheek. Physical examination revealed a 1 cm firm subcutaneous nodule overlying the right masseter. Due to concerns about deep connections to underlying structures, the procedure was deferred at this time, and the patient elected to follow up with oncology for further imaging.

## Discussion

RPLs are generally associated with a poor prognosis due to their aggressive nature and ability to metastasize. While the literature supports metastatic disease in high-grade RPL, we present a novel case highlighting cutaneous metastatic disease in a low-grade RPL, thereby challenging established paradigms. Although low-grade RPLs are typically prognostically favorable, the presence of metastasis, even from a low-grade primary, is associated with a significantly increased risk of mortality [7]. Factors that may contribute to metastasis in these low-grade tumors include their location, as in this patient, whose tumor caused mass effect on her inferior vena cava, with which direct contact could have provided a rapid way for the tumor to disseminate despite its low histologic grade. Furthermore, the gross size of the lesion may be linked to its aggressive behavior and metastatic ability, despite its low histologic grade. Other factors may include common genetic mutations observed in leiomyosarcomas, among them being *PTEN*, *RB1*, and *TP53* [8]. With all of these other potential factors in mind, we urge clinicians to carefully consider the prognostic significance of low histologic grade in such tumors. Additionally, for patients who undergo surgical resection, as in this case, the most significant predictors for mortality include tumor size, tumor location, and mitotic rate [9].

In any case, close multidisciplinary follow-up is vital to the overall health and well-being of these patients. The healthcare team must work together to manage these complex cases and should include, but not be limited to, surgery, oncology, dermatology, and mental health resources. For isolated cutaneous metastasis, surgical excision is preferred in addition to palliative care. For widespread metastatic lesions, systemic therapy combining doxorubicin with either dacarbazine or ifosfamide remains first-line. However, other systemic agents have been noted in the literature, including but not limited to methotrexate, trabectedin, and olaratumab [10,11]. However, it is important to note that systemic options are limited and their efficacy is not well supported in low-grade leiomyosarcoma. Ultimately, in patients with a history of low-grade RPL presenting with new-onset skin lesions, it is imperative to maintain a high index of suspicion for metastatic disease to provide appropriate and effective care.

## Conclusions

Cutaneous metastasis of RPL is a recognized phenomenon in the literature; however, these metastases are typically high-grade, aggressive tumors. In contrast, we present a novel case of low-grade primary RPL metastasizing to the skin. Clinical findings were non-specific, but histopathologic examination revealed characteristic findings of a low-grade leiomyosarcoma with fewer than seven mitotic figures per 10 HPF and a Ki-67 index of 7%. En bloc resection remains the mainstay of therapy for RPL, with surgical excision preferred for cutaneous metastases. Adjuvant chemotherapeutic agents and radiation have been discussed in the literature, but no conclusive evidence has been found to support their use. We emphasize that low-grade differentiation should not preclude clinicians from being suspicious of metastatic disease in patients with RPL. Thus, multidisciplinary follow-up and ongoing vigilance are crucial for a thorough workup of metastasis in patients with low-grade RPL and the development of new skin lesions.

## References

[1] Hermi A, Boussaffa H, Saadi A, BelHadjKacem L, Chakroun M, Slama RB (2023). Giant retroperitoneal leiomyosarcoma: a case report. J Surg Case Rep.

[2] Alektiar KM (2021). Soft-tissue sarcoma. Gunderson & Tepper’s Clinical Radiation Oncology.

[3] Grimaudo MS, Renne SL, Colombo P (2024). Prognostic value of mitotic count in leiomyosarcoma: a comprehensive monocentric retrospective study. Hum Pathol.

[4] D'Ambrosio L, Van Houdt W, Stelmes JJ, Gronchi A (2022). First and further-line multidisciplinary treatment of retroperitoneal sarcomas. Curr Opin Oncol.

[5] Tan MC, Brennan MF, Kuk D (2016). Histology-based classification predicts pattern of recurrence and improves risk stratification in primary retroperitoneal sarcoma. Ann Surg.

[6] Soares Queirós C, Filipe P, Soares de Almeida L (2021). Cutaneous leiomyosarcoma: a 20-year retrospective study and review of the literature. An Bras Dermatol.

[7] Paal E, Miettinen M (2001). Retroperitoneal leiomyomas. A clinicopathologic and immunohistochemical study of 56 cases with a comparison to retroperitoneal leiomyosarcomas. Am J Surg Pathol.

[8] Cope BM, Traweek RS, Lazcano R, Keung EZ, Lazar AJ, Roland CL, Nassif EF (2023). Targeting the molecular and immunologic features of leiomyosarcoma. Cancers (Basel).

[9] Abraham JA, Weaver MJ, Hornick JL, Zurakowski D, Ready JE (2012). Outcomes and prognostic factors for a consecutive case series of 115 patients with somatic leiomyosarcoma. J Bone Joint Surg Am.

[10] Winchester DS, Hocker TL, Brewer JD (2014). Leiomyosarcoma of the skin: clinical, histopathologic, and prognostic factors that influence outcomes. J Am Acad Dermatol.

[11] Kazlouskaya V, Lai YC, Khachemoune A (2020). Leiomyosarcoma of the skin: review of the literature with an emphasis on prognosis and management. Int J Dermatol.

